# DAMFCMI: capturing cross-view interactions via hybrid attention for circRNA–MiRNA interaction prediction

**DOI:** 10.1093/bioadv/vbag109

**Published:** 2026-07-20

**Authors:** Tao Bai, Xiupan Ma, Lanlan Sun, Zongwen Bai, Wendong Wang, Hang Wei

**Affiliations:** School of Mathematics and Computer Science, Yan’an University, Yan’an, Shaanxi 716000, China; School of Mathematics and Computer Science, Yan’an University, Yan’an, Shaanxi 716000, China; School of Mathematics and Computer Science, Yan’an University, Yan’an, Shaanxi 716000, China; School of Physical and Electronic Information, Yan’an University, Yan’an, Shaanxi 716000, China; School of Mathematics and Computer Science, Yan’an University, Yan’an, Shaanxi 716000, China; School of Computer Science and Technology, Xidian University, Xi’an, Shaanxi 710126, China

## Abstract

**Motivation:**

Circular RNAs (circRNAs) and microRNAs (miRNAs) play pivotal roles in gene expression regulation, where understanding their interactions (CMIs) is essential for deciphering the molecular mechanisms behind cellular physiological and pathological states. Most existing approaches to CMI prediction are constrained by their reliance on shallow, single-view representations, while deep models typically align only on final embeddings, thereby neglecting the rich layer-wise interactions that are critical for capturing biological complexity.

**Results:**

To address these issues, we propose DAMFCMI, a novel method for CMI prediction. DAMFCMI characterizes circRNAs and miRNAs through three distinct feature views: sequence-based, attribute-based, and behavior-based features. A hybrid attention mechanism captures dependencies within individual views through multi-head self-attention and across views through cross-attention, enabling comprehensive modeling of feature interactions. Experimental results show that DAMFCMI outperforms state-of-the-art methods across three benchmark datasets. Visualization analyses demonstrate that the hybrid attention architecture enhances feature discriminability through effective multi-view feature integration. Moreover, case studies show that 13 out of 15 predicted CMIs predicted by DAMFCMI are supported by evidence in the PubMed literature, underscoring its potential for uncovering biologically relevant interactions.

**Availability and implementation:**

The data and source code are available at https://github.com/yadxbiolab/DAMFCMI

## 1 Introduction

Circular RNAs (circRNAs) and micro RNAs (miRNAs) are two major classes of non-coding RNAs, with circRNAs forming covalently closed loops that confer high stability (Cech and Steitz 2014, Jeck and Sharpless 2014, Salzman 2016), and miRNAs functioning as post-transcriptional regulators of gene expression (He and Hannon 2004, Wang *et al.* 2023). Accumulating evidence has demonstrated that circRNAs can function as competing endogenous RNAs (ceRNAs) by acting as molecular sponges for miRNAs, participating in post-transcriptional gene regulation (Hansen *et al.* 2013; Memczak, *et al.* 2013; Zhang, *et al.* 2020). The circRNA–miRNA associations play a pivotal role in numerous physiological and pathological processes, including various human diseases such as cancer, cardiovascular disorders, and neurological conditions (Qi *et al.* 2015, Zhong *et al.* 2018, Patop *et al.* 2019, Chen *et al.* 2021). For example, Zhuang *et al.* (2020) found that *hsa_circ_0075828* promoted bladder tumor cell proliferation by sponging miR-1224-5p, leading to deregulation of CREB1 expression. Shao *et al.* (2020) demonstrated that *hsa_circ_0075341* facilitated cervical cancer cell proliferation and invasion by sponging miR-149-5p, thereby modulating the AURKA signaling axis. Identifying circRNA-miRNA interactions (CMIs) is critical to understanding disease-driving molecular mechanisms, enhancing genetic regulation studies, and therapeutic advancement (Zhang *et al.* 2017, Yi *et al.* 2019).

Although traditional biological experiments provide reliable validation of circRNA-miRNA association (Xiong *et al.* 2018, Fang and Lei 2019, Tong *et al.* 2022, Wang *et al.* 2023), these methods are often time-consuming, labor-intensive, and low-throughput, which limits their feasibility for large-scale and systematic studies. In recent years, the rapid accumulation of CMI data has led to the development of numerous computational models (Dori and Bicciato 2019). To consolidate advances in this field, Chen *et al.* (Wang *et al.* 2021, Huang *et al.* 2022a, 2022b, 2022c) have systematically summarized the biogenesis and functions of circRNAs and miRNAs, key database resources, and the development of computational prediction models.

Existing computational methods for CMI prediction can generally be categorized into three groups. The first category emphasizes single-view feature models that primarily rely on similarity matrices derived from known circRNA–miRNA associations. For example, NECMA (Lan *et al.* 2021) constructs circRNA and miRNA similarity matrices using Gaussian interaction profile kernels, integrates them into a heterogeneous network, and applies the NetMF algorithm for network embedding. The second category incorporates multi-source features to enhance the richness and informativeness of the data. However, most methods consider only two types of features and utilize shallow fusion strategies—such as direct concatenation—thereby limits the ability to capture dependencies across feature modalities. For example, CMIVGSD (Qian *et al.* 2021) combines linear features obtained via singular value decomposition with non-linear embeddings from variational graph autoencoders, and employs LightGBM for classification, and BGF-CMAP (Guo *et al.* 2024a, 2024b) fuses semantic embeddings derived from Word2Vec with topological information extracted using LINE and Graph Factorization, followed by a gradient-boosted decision tree classifier. The third category enriches feature representations by incorporating intrinsic sequence information. For instance, KGDCMI (Wang *et al.* 2022) integrates Gaussian kernel similarity, K-mer-based embeddings, and graph-structured features within a deep neural network framework. To address this limitation, BCMCMI (Wei *et al.* 2023) addresses this limitation by employing BERT to extract high-level semantic features from sequences and Metapath2vec to encode topological relationships.

Although previous methods have achieved promising results, several critical challenges remain to be addressed: (i) Insufficient exploitation of sequence features. Most existing methods overlook the intrinsic sequence information and physicochemical features of circRNAs and miRNAs, leading to a failure in capturing their intrinsic biological patterns. (ii) Limited diversity of feature views. Most current approaches rely on only one or two types of feature information, constraining their capacity to fully exploit the complementary advantages offered by diverse biological perspectives. (iii) Shallow fusion of heterogeneous features. Existing methods neglect the exploration of deep collaborative features in the interactions between circRNA and miRNA, and adopt shallow fusion strategies such as simple splicing or weighted averaging in fusing multi-view features, thereby lacking the ability to perform deep feature extraction across distinct views.

To solve the above limitations, we propose a hybrid attention-guided multi-view feature fusion framework, named DAMFCMI, for identifying CMIs. DAMFCMI incorporates three biologically meaningful and complementary features from different perspectives: attribute features that capture local contextual patterns, behavioral features derived from graph embeddings to represent global interaction dependencies, and sequence features that reflect intrinsic biological information and physicochemical properties of molecules. The hybrid attention module, including multi-head self-attention (MSA) and cross-attention (CA), is designed to effectively capture contextual dependencies within individual features and complex correlations across different feature types, enabling dynamic and hierarchical fusion of semantic attributes, topological behaviors, and sequence-level information. Experiments on three publicly available benchmark datasets demonstrate that DAMFCMI significantly outperforms state-of-the-art methods in both predictive performance and generalization ability. The effectiveness of the multi-view feature fusion strategy and dependency extracting mechanism is further validated through a series of ablation and comparative analyses. In case study, 12 out of the 15 predicted potential CMIs were supported by existing literature. The source code and data are available on https://github.com/yadxbiolab/DAMFCMI.

## 2 Methods

### 2.1 Datasets

To ensure comparability with existing studies, we follow the standard settings in previous work (Guo *et al.* 2024a, 2024b) for constructing positive and negative samples as well as for data preprocessing, and maintain a 1:1 ratio between positive and negative samples. All datasets originate from two commonly used public databases: CircR2Cancer (Lan *et al.* 2020) and CircBank (Liu *et al.* 2019). A summary of the dataset statistics is provided in Table 1.

**Table 1:** Elaborate data regarding CMIs employed in DAMFCMI.

Dataset	CMI-20208	CMI-9589	CMI-9905
CircRNA	3569	2115	2346
MiRNA	1152	821	962
Interactions	20208	9589	9905

### 2.2 Method overview

In this study, we propose a hybrid-attention-guided multi-view feature fusion framework, named DAMFCMI, for CMIs prediction. The overall process of DAMFCMI is shown in Fig. 1 with three parts: (i) low-dimensional vector embedding (Fig. 1A). Feature representations of circRNAs and miRNAs are extracted from multiple perspectives using diverse strategies, including intrinsic physicochemical sequence features, attribute features, and interaction behavior features; (ii) hybrid-attention module for deep feature fusion (Fig. 1B). MSA captures local dependencies within individual feature types, while CA captures global interactions across different feature views; (iii) CMIs prediction (Fig. 1C). The fused features from all sources are concatenated and passed through a fully connected layer to predict the probability of interaction for each circRNA–miRNA pair, thereby determining whether it constitutes a valid CMIs.

**Figure 1:**
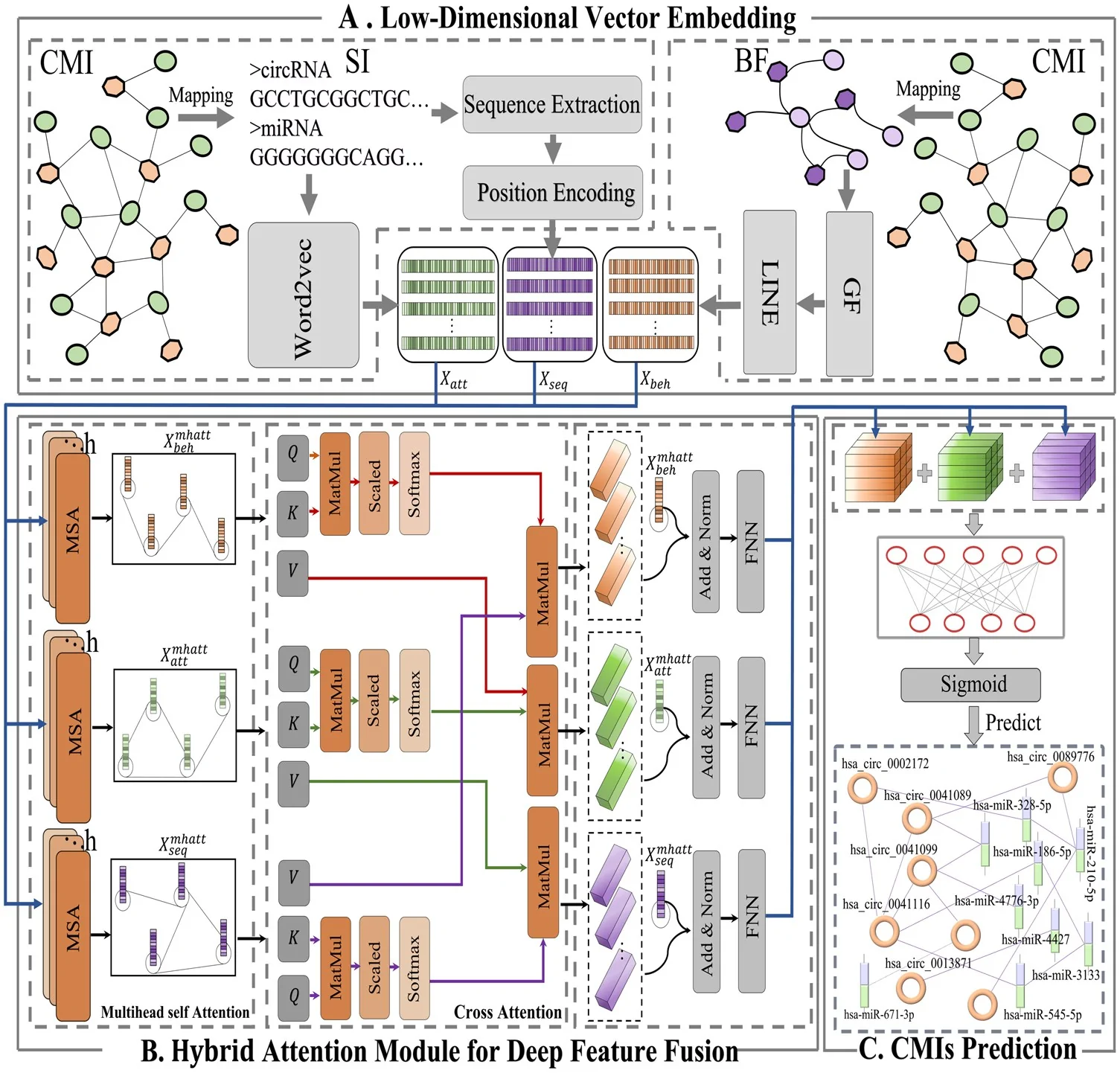
The workflow of the DAMFCMI.

### 2.3 Multi-view feature extraction

To comprehensively characterize circRNAs and miRNAs, we propose a multi-view feature extraction strategy that integrates sequence features, attribute features, and interaction behavior information. Specifically, circRNA and miRNA sequences are encoded to generate semantic embeddings, which serve as attribute features. In parallel, a homogeneous interaction graph is constructed based on known associations, and graph embedding techniques are applied to capture topological behavioral features that reflect potential interaction patterns. Furthermore, to overcome the limitations of prior studies that overlooked inter-nucleotide dependencies, we extract the intrinsic sequence information and physicochemical features directly from raw sequences as supplementary sequence features.

#### 2.3.1 Sequence feature extraction

To address the insufficient exploitation of sequence features, a set of sequence features directly from the raw sequences is extracted (Wang *et al.* 2023).

These features were designed to capture diverse aspects of the sequences, including coding potential, compositional patterns, and physicochemical properties. Specifically, seven types of descriptors were included: open reading frame length (ORF-length) (Liu *et al.* 2019), open reading frame coverage (ORF-coverage) (Chen *et al.* 2021), Hexamer score, Fickett score, composition–transition–distribution (CTD) (Meinhart *et al.*2005), isoelectric point (pI), and electron–ion interaction potential (EIIP) (Finkel *et al.* 2018), as detailed in Table 2.

**Table 2:** The detailed information on the different features used in DAMFCMI.

Feature descriptor	Dimension	Feature type
ORF-length	1	Intrinsic sequence information
ORF-coverage	1	
Hexamer score	4	
Fickett score	2	
CTD	30	
pI	2	Physicochemical property
EIIP	8	

#### 2.3.2 Attribute feature extraction by word2vec

Inspired by the concept of Word2Vec in natural language processing (Li *et al.* 2021, Cao *et al.* 2025), Word2Vec is used to capture the latent semantic information embedded in circRNA and miRNA nucleotide sequences (Church 2017). Each nucleotide is treated as word, while the entire sequence is viewed as a sentence composed of these words arranged in a specific order (Guo *et al.* 2024a, 2024b). The continuous bag-of-words (CBOW) architecture in Word2Vec is adopted to predict each target nucleotide based on its surrounding context. It can be denoted as (Guo *et al.* 2022):


(1) LCBOW=1N∑i=1NlogP(wi|Ci)

where $w_{i}$ is the current target base to be predicted, $C_{i}=\{w_{i-c,}\ldots ,w_{i-1,}w_{i+1,}\ldots ,w_{i+c,}\}$ is the set of context bases of the target base $w_{i}$, c is the size of the context window, and N is the total number of bases in the sequence. This method generates a 64-dimensional embedding vector.

#### 2.3.3 Behavior feature extraction by homogeneous graph embedding

Two types of homogeneous graph embedding methods (Guo *et al.* 2024a, 2024b) are employed to investigate the behavioral characteristics of circRNA and miRNA within the interaction network. The first is graph factorization (GF) (Ahmed *et al.* 2013), which relies on matrix decomposition. The GF approximates the adjacency matrix of the graph by mapping the connection strength between nodes to the inner product of their embedding vectors, thereby preserving local structural relationships within the network (Li *et al.* 2021). The optimization objective of GF can be defined as minimizing a combination of the reconstruction error and a regularization term:


(2) minZ⁡∑(u,v)∈ε(Auv-UuUv)2+λ∑u∈v‖Zu‖2

where $A_{uv}$ is the connection weight between node u and node v in the adjacency matrix of the graph, Z and λ are the embedding matrix and regularization coefficient, respectively.

The second is Large-scale Information Network Embedding (LINE) based on depth modeling (Goyal and Ferrara 2018), which is oriented to large-scale networks and captures first-order adjacencies and second-order similarities between nodes, respectively, thus capturing both local and global structural features. Its objective functions for first-order similarity and second-order similarity are, respectively:


(3) L1=-∑(u,v)∈εwuv⋅logσ(zuTzv)

(4) L2=-∑(u,v)∈εwuv⋅logσ(zv'Tzu)

where $w_{uv}$ is the weight of edge(u,v), $z_{u}$ and $z_{v}$ are the embedding vectors of nodes u and v, σ(·) is the sigmoid function. After the above two feature processing steps, each circRNA and miRNA node is ultimately embedded into a unified 64-dimensional space as its behavioral feature representation.

### 2.4 Hybrid attention module for deep feature fusion

#### 2.4.1 Multi-head self-attention

Features from different views often vary significantly in dimension and representation, posing challenges for capturing contextual dependencies and global semantic relationships. To effectively capture the long-range dependencies within each type of feature, we adopt the MSA mechanism. This mechanism captures deep contextual information within sequence features, attribute features, and behavioral features by attending to different representation subspaces in parallel (Zhao *et al.* 2022). Specifically, the MSA mechanism assigns multiple heads of attention to each input feature vector separately, enabling each attention head to capture distinct aspects of each input (Du *et al.* 2023). For each head, an attention distribution is computed by linearly projecting the input to obtain query (*Q*), key (*K*), and value (*V*) matrices. The attention can be summarized as follows (Wang *et al.* 2024, 2025):


(5) Q=XWQ∈Rd×dk,K=XWK∈Rd×dk,V=XWV∈Rd×dV

where $W^{Q},W^{K},W^{V}$ are the trainable weight matrices, $d_{k}$=$d_{V}$=d/h is the subspace dimension of each head.

The attention distribution of each head will be used to weight and sum the corresponding value vector to get the output of that head (Wang *et al.* 2024):


(6) Attention(Q,K,V)=softmax(QKTdk)V

The final representation is obtained by concatenating the outputs of all attention heads, followed by a linear transformation:


(7) MultiHead(Q,K,V)=Concat(head1,…,headh)WO

where each individual attention head computes:


(8) headi=Attention(QWiQ,KWiK,VWiV)

where h is the number of attention heads, i∈{1,2,…,h}, and $W^{O}$is the linear transformation weight matrix of the output.

#### 2.4.2 Cross-attention network

Traditional fusion methods, such as simple concatenation or weighted summation, often fall short in capturing the deep dependencies and complex interactions among these heterogeneous features. To capture the intricate interactions between circRNA and miRNA features derived from different views, we introduce a CA mechanism (Liu *et al.* 2024). The core strength of CA mechanisms lies in their capacity to explicitly capture information-driven dependencies across heterogeneous modalities. In our framework, we construct pairwise CA modules among the three feature types, as defined below (Lu *et al.* 2025):


(9) Qi=XiWiQ, Ki=XiWiK, Vi=XiWiV

where $X_{i}\in R^{n_{i}\times d}$ is the input representation of the ith views, with i∈{att,beh,seq} indicating attribute, behavior, and sequence feature. $W_{i}^{Q},W_{i}^{K}\in R^{d\times d_{k}}$ and $W_{i}^{V}\in R^{d\times d_{v}}$are learnable projection matrices are specific to each view.

To estimate the cross-view association between attribute and behavior features, we calculate the dot product between the query matrix of $X_{\mathrm{att}}$ and the key matrix of $X_{\mathrm{beh}}$. Specifically, given a query representation from the attribute modality and key–value representations from the behavioral modality, the CA mechanism is computed as follows:


(10) CrossAttention(att←beh)=softmax(QattKbehTdk)Vbeh

where softmax(·) denotes the softmax function, which normalizes raw logits into a valid probability distribution over the target classes.$\;d_{k}$ is the dimensionality of the key vectors (Wang *et al.* 2024), used to scale the dot-product for numerical stability.

#### 2.4.3 Hybrid-attention fusion module

After obtaining node representations through the MSA and CA mechanisms, the hybrid-attention-driven feature fusion framework is designed to preserve the complementary information captured by both attention branches while stabilizing the training process (Zhai *et al.* 2020, Zhao *et al.* 2024). To this end, an effective fusion strategy is proposed to enhance the representation quality of the extracted heterogeneous features. The strategy combines the multi-attribute features from both mechanisms through element-wise addition, followed by layer normalization. Formally, we fuse the outputs of the MSA and CA modules via element-wise addition, followed by layer normalization to maintain information fidelity and promote robust feature learning (Liu *et al.* 2024). The fusion process is computed as follows:


(11) Xi=LayerNorm(Ximhatt+Xicross), i∈{att,beh,seq}

where $X_{i}^{\mathrm{mhatt}}$ is output of the MSA mechanism for the ith views, while $X_{i}^{\mathrm{cross}}$ denotes the ith views output of the CA mechanism for the same views.

Following the completion of feature interaction through the hybrid attention mechanisms, the fused features are further processed by a feed-forward neural network (FFN) to enhance nonlinear representational capability. The FFN consists of two fully connected layers with a nonlinear activation function in between (Moller 1997), and its computation is formally defined as:


(12) FFN(Xi)=Relu(XiW1+b1)W2+b2

where $X_{i}$ is the ith output feature of fusion strategy module, Relu(·) is the Relu activation function, $W_{1}\;\mathrm{and}\;b_{1}$ are the weights and biases of the first layer, and $W_{2}\;\mathrm{and}\;b_{2}\;$are the weights and biases of the second layer.

### 2.5 Association prediction

After completing the deep fusion of multi-source features, the resulting representations are concatenated to form a unified feature vector. This unified representation is then passed through a fully connected layer to capture nonlinear relationships and enhance the expressive power of the model, which is defined as follows:


(13) Ffused=[Fseq∥Fatt∥Fbeh]

(14) h=σ(WFfused+b)

where ∥ denotes the concatenation operation, Wand b are the learnable weights and bias parameters, and σ represents a nonlinear activation function.

To predict the likelihood of potential interactions between circRNAs and miRNAs, a sigmoid activation function is applied to the output:


(15) y^=11+e-(h+b)

where $\hat{y}\in [0,1]$ represents the predicted interaction probability, W denotes the weight matrix and b is the bias of the final linear layer.

### 2.6 Evaluation metrics

Seven metrics, including Accuracy (Acc), Sensitivity (Sen), Specificity (Spe), Precision (Prec), Matthews Correlation Coefficient (MCC), Area Under the Precision–Recall Curve (AUPR), and Area Under the Receiver Operating Characteristic Curve (AUC) (Wang *et al.* 2020, 2023, Li *et al.* 2024) are used to evaluate the predictive performance. The definitions of these metrics are calculated by:


(16) ACC=TP+TNTP+TN+FP+FN

(17) Sen=TPTP+FN

(18) Spe=TNTN+FP

(19) Prec=TPTP+FP

(20) MCC=TP·TN-FP·FN(TP+FP)(TP+FN)(TN+FP)(TN+FN)

where TP, TN, FP, and FN represent the number of true positives, true negatives, false positives, and false negatives, respectively.

## 3 Experiments and results

### 3.1 Head number optimization in multi-head attention

The MSA mechanism captures contextual dependencies within each feature view, with the number of attention heads serving as a critical hyperparameter that influences the ability to attend to distinct representation subspaces. To evaluate its effect, comparative experiments were conducted using 2, 4, 6, 8, and 10 attention heads. The experimental results of DAMFCMI with different head numbers are shown in Table 3.

**Table 3:** Evaluate the impact of the number of heads in multi-head attention on the DAMFCMI.

Heads	ACC	Sen	Spe	Prec	MCC	AUPR	AUC
10	0.8980	0.8913	0.9046	0.9038	0.7964	0.9585	0.9593
8	**0.9077**	0.8955	**0.9199**	**0.9180**	**0.8159**	**0.9629**	**0.9623**
6	0.9020	0.8943	0.9097	0.9084	0.8043	0.9578	0.9581
4	0.9014	0.8953	0.9074	0.9064	0.8029	0.9595	0.9599
2	0.9045	**0.9104**	0.8986	0.9002	0.8094	0.9613	0.9615

The best performance of each metric is highlighted in bold.

From which we can see the following: (i) When using a smaller number of heads, the DAMFCMI shows slightly reduced accuracy and discriminative power. This may be due to the limited capacity of fewer heads to capture diverse feature patterns, thereby restricting the capture of fine grained dependencies across different modalities. (ii) Conversely, increasing the number of heads beyond eight does not lead to further performance gains and even causes a slight decline. This degradation is likely due to over-fragmentation of the feature space, where each attention head receives less informative input, thereby diminishing the effectiveness of the attention mechanism. Furthermore, an increased number of heads introduces additional computational overhead and optimization challenges. (iii) The best overall performance is achieved when the number of attention heads is set to 8, striking a balance between representational capacity and computational efficiency.

### 3.2 Prediction performance of DAMFCMI

To comprehensively evaluate the classification performance and generalization capability of DAMFCMI, five-fold cross-validation was performed on three benchmark datasets: CMI-20208, CMI-9589, and CMI-9905, with the detailed results presented in Table 4.

**Table 4:** Experimental results on the CMI-20208, CMI-9589, and CMI-9905 datasets.

Datasets	ACC	Sen	Spe	Prec	MCC	AUPR	AUC
CMI-20208	0.9077	0.8955	0.9199	0.9180	0.8159	0.9629	0.9623
CMI-9905	0.9335	0.9440	0.9230	0.9248	0.8674	0.9746	0.9799
CMI-9589	0.9324	0.9299	0.9349	0.9349	0.8651	0.9773	0.9801

It can be observed that DAMFCMI consistently achieves robust and superior performance across all datasets. Specifically, DAMFCMI effectively identified both positive and negative CMIs with high accuracy. On the CMI-9905 and CMI-9589 datasets, DAMFCMI achieved the highest accuracy scores of 0.9335 and 0.9324, as well as AUC values of 0.9799 and 0.9801. This can be attributed to the higher AUPR and AUC in these two datasets, enabling the framework to fully exploit the multi-source features of the input. In contrast, on CMI-20208, while the dataset size is larger and potentially noisier, DAMFCMI still demonstrated strong classification capability on this dataset, achieving an AUC of 0.9623 and an AUPR of 0.9629. These findings suggest that the integration of sequence, attribute, and behavioral features through MSA and CA mechanisms effectively enhances the ability to extract informative signals from sparse interaction data.

Moreover, the ROC and PR curves obtained by DAMFCMI across all three datasets (Fig. 2) demonstrate high inter-fold consistency with minimal standard deviation, confirming the stability and reproducibility of DAMFCMI for CMI prediction. The detailed five-fold cross-validation results for other metrics are provided in Tables 4–6, available as supplementary data at *Bioinformatics Advances* online.

**Figure 2:**
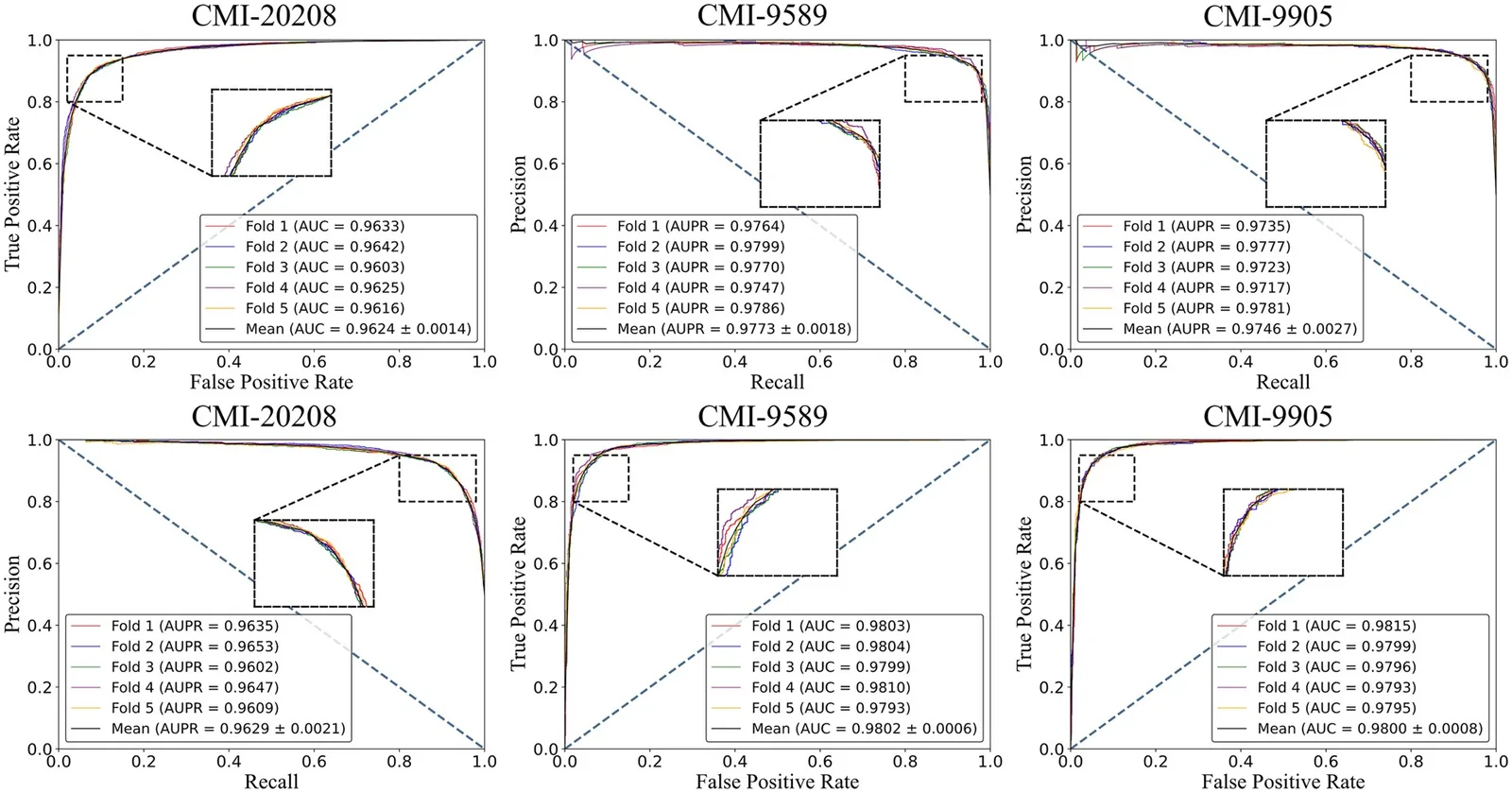
Assessment of DAMFCMI’s discriminative power and categorical balance.

**Table 5:** Evaluating the performance of various methods on Dataset-20208, Dataset-9589, and Dataset-9905.

Datasets	CMI-20208			CMI-9589			CMI-9905	
Methods	AUPR	AUC	Methods	AUPR	AUC	Methods	AUPR	AUC
WSCD	0.8847	0.8898	KGDCMI	0.8937	0.9041	KGDCMI	0.8767	0.8930
KGDCMI	0.8767	0.8930	CACMA	0.9086	0.9156	SGCNCMI	0.8887	0.8942
SGCCMI	0.8887	0.8942	KS-CMI	0.9181	0.9179	JSNDCMI	0.8999	0.9033
BGF-CMAP	0.9120	0.9075	GCNCMI	0.9396	0.9320	KS-CMI	0.9144	0.9086
CA-CMA	0.9131	0.9170	JSNDCMI	0.9403	0.9415	CA-CMA	0.9088	0.9138
LSAGHI	0.9479	0.9522	LSAGHI	0.9520	0.9552	LSAGHI	0.9435	0.9405
DGCLCMI	0.9415	0.9546	DGCLCMI	0.9501	0.9610	DGCLCMI	0.9546	0.9645
MFERL	0.9443	0.9456	MFERL	0.9629	0.9669	MFERL	0.9610	0.9596
MuGNetCMI	0.9187	0.9221	MuGNetCMI	0.9577	0.9568	MuGNetCMI	0.9278	0.9248
iHofman	0.9211	0.9173	iHofman	0.9269	0.9187	iHofman	0.9234	0.9095
**Ours**	**0.9629**	**0.9623**	**Ours**	**0.9773**	**0.9801**	**Ours**	**0.9746**	**0.9799**

The best performance of each metric is highlighted in bold.

**Table 6:** Performance evaluation of different feature combinations.

Ablation settings			ACC	Sen	Spe	Prec	MCC	AUPR	AUC
Att	Beh	Seq							
✓	✗	✗	0.6798	0.6558	0.7039	0.6889	0.3602	0.7372	0.7368
✓	✓	✗	0.9010	0.8931	0.9090	0.9081	0.8027	0.9589	0.9596
✓	✓	✓	**0.9077**	**0.8955**	**0.9199**	**0.9180**	**0.8159**	**0.9629**	**0.9623**

The best performance of each metric is highlighted in bold, the symbol ✗ denotes exclusion of the module, while ✓ denotes inclusion.

### 3.3 Performance comparison with competing methods

To comprehensively evaluate the performance of DAMFCMI, we systematically compared it with several state-of-the-art CMI prediction methods on three benchmark datasets. The first category of methods mainly relies on sequence and similarity features in combination with traditional machine learning model, such as CMIVGSD (Qian *et al.* 2021), BGF-CMAP (Guo *et al.* 2024a, 2024b), JSNDCMI (Wang *et al.* 2023), and KS-CMI (Wang *et al.* 2023). The second category focuses on graph representation learning and graph neural networks to capture the topological information between circRNAs and miRNAs. Representative methods in this category include GCNCMI (He *et al.* 2022), SGCNCMI (Yu *et al.* 2022), KGDCMI (Wang *et al.* 2022), and LSAGHI (Yin *et al.* 2025). The third category integrates techniques such as Word2Vec, LSTM, or autoencoders to represent multi-source features and employs deep neural networks to predict circRNA–miRNA associations. In addition, some methods further incorporate attention mechanisms, heterogeneous network structures, or contrastive learning strategies to enhance the representation of both local and global structural information. Representative methods include WSCD (Guo *et al.* 2022), CA-CMA (Guo *et al.* 2024a, 2024b), DGCLCMI (Cao *et al.* 2025), iHofman (Yu *et al.* 2025), MFERL (Peng *et al.* 2025), and MuGNet-CMI (Jiang et al. 2025).

To ensure fair and consistent comparisons, the experimental results of all baseline methods were directly obtained from their original publications and reported under the same dataset settings. Table 5 illustrates that DAMFCMI outperformed all baseline methods, achieving the highest AUPR and AUC across all three datasets. On the CMI-9905 dataset, DAMFCMI attained AUC and AUPR exceeding 0.97, representing improvements of 1.6% and 1.42%, respectively, over the best-performing baseline method, MFERL. Similarly, on the CMI-9589 dataset, DAMFCMI outperformed MFERL by 1.37% in AUC and 1.50% in AUPR. Figure 3A–3C shows the corresponding comparative performance via two-indicator radar charts, where each polygon represents the distribution of AUC or AUPR across all compared methods. On all three datasets, DAMFCMI occupies the outermost positions in both polygons, clearly demonstrating its superiority under both evaluation criteria. To further validate the statistical significance of these improvements, we conducted additional experiments on the CMI-20208 dataset, with the statistical test results presented in Table 3, available as supplementary data at *Bioinformatics Advances* online.

**Figure 3:**
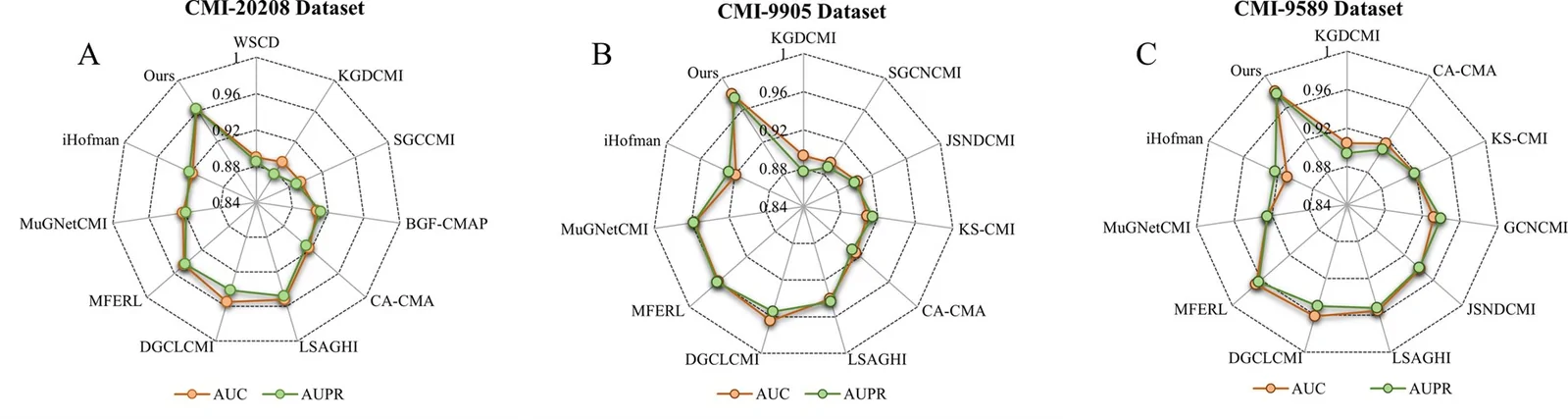
Performance comparison with various methods on three CMI datasets.

### 3.4 Multi-view feature fusion contributes to representation enhancement

To investigate whether fusing multi-view features enhances the representational capacity, we conducted feature ablation studies on the CMI-20208 dataset, with the specific settings as follows: (i) using only attribute features (Att); (ii) fusing attribute and behavioral features (Att + Beh); (iii) integrating attribute, behavioral, and sequence features (Att + Beh + Seq).

As summarized in Table 6 and Fig. 4, the method exhibited the weakest performance when only attribute features were used, achieving an accuracy of 67.98% and an AUC of 0.7368. Upon incorporating behavioral features, the performance improved substantially, with increases of 32.54% in accuracy, 36.18% in sensitivity, and over 30% in both AUPR and AUC, along with a remarkable 122.85% increase in MCC. This underscores the significant contribution of behavioral features in enhancing the capture of interaction patterns. When sequence features were further incorporated, the model achieved the best performance across all metrics, with improvements of 1.2% in Spe, 1.09% in Prec, and 1.64% in MCC. These results demonstrate the importance of multi-view feature fusion, as sequence information captures unique biological characteristics.

**Figure 4:**
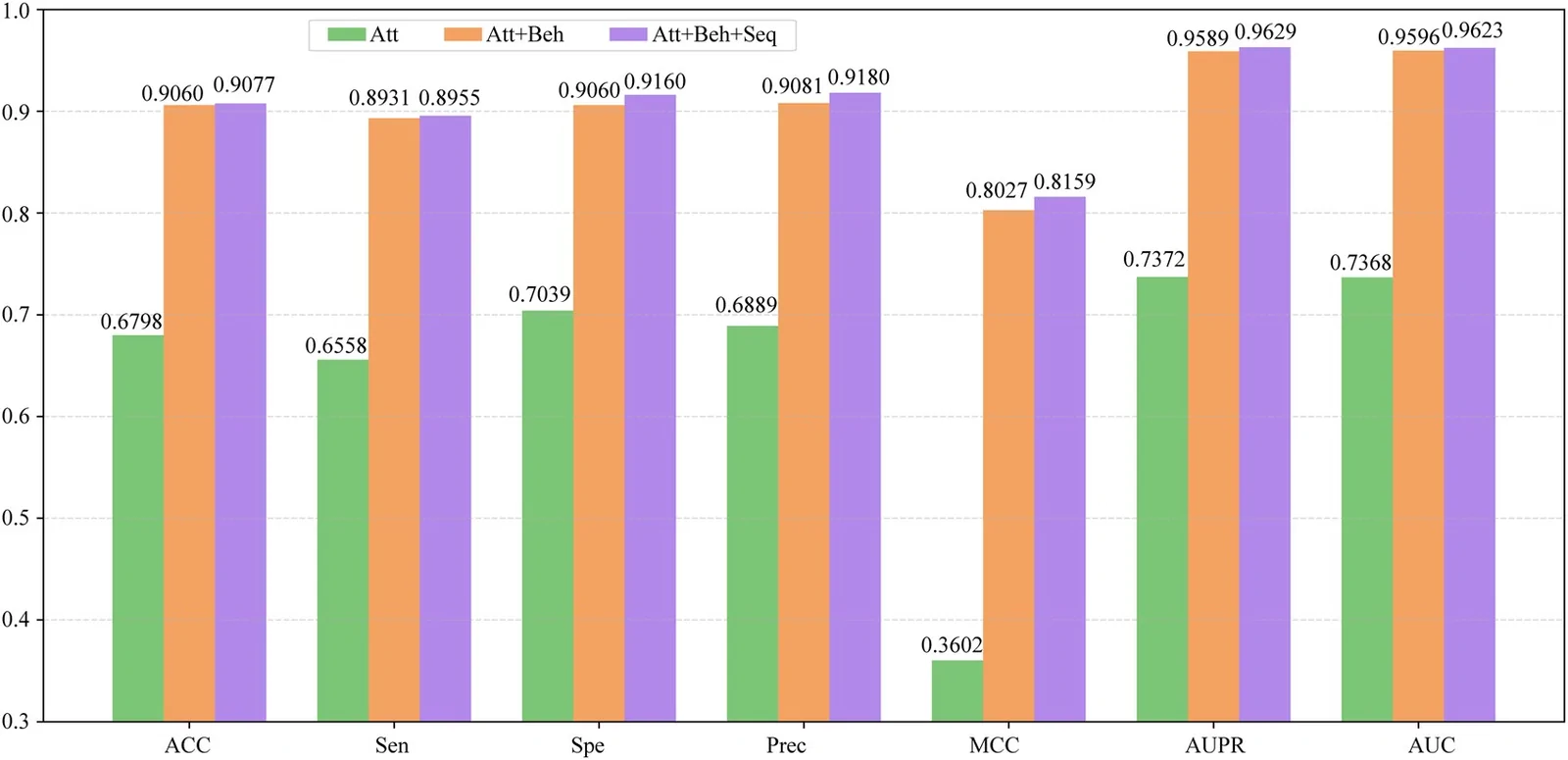
Comparison of predictive ability for different combinations of features.

### 3.5 Hybrid attention mechanism synergistically improves prediction performance

To investigate the contribution of individual components in DAMFCMI, we conducted ablation experiments on the CMI-20208 dataset, evaluating four DAMFCMI variants. Three of these variants were created by selectively removing key components—the Multi-Head Self-Attention (MSA) and Cross-Attention (CA) mechanisms. The results are presented in Table 7. When both MSA and CA modules are removed, DAMFCMI exhibits the lowest performance across all evaluation metrics. When only the MSA module is retained, the performance notably improves, with Prec and MCC increasing by 1.95% and 2.63%, ACC increasing by 1.16%, respectively, compared to the setting without either module. This confirms that the attention mechanisms are essential for capturing complex feature dependencies. Interestingly, although the configuration with only MSA achieves the highest in terms of AUC and AUPR, it underperforms on five other metrics—including ACC and MCC—indicating that MSA alone cannot ensure balanced performance. When only the CA module is included, the model also shows moderate improvements compared to the baseline. However, it still lags behind the full DAMFCMI configuration. In contrast, the complete DAMFCMI, integrating both MSA and CA, achieves the best performance. The results show that although both MSA and CA individually improve performance, their integration in DAMFCMI achieves the best overall results by jointly modeling contextual dependencies and cross-modal feature interactions.

**Table 7:** Performance evaluation of different module combinations of the DAMFCMI.

Ablation settings		ACC	Sen	Spe	Prec	MCC	AUPR	AUC
CA	MSA							
✗	✗	0.8928	0.8920	0.8938	0.8937	0.7859	0.9581	0.9570
✗	✓	0.9032	0.8938	0.9126	0.9111	0.8066	**0.9639**	**0.9635**
✓	✗	0.8958	0.8913	0.9057	0.9046	0.7974	0.9587	0.9586
✓	✓	**0.9077**	**0.8955**	**0.9199**	**0.9180**	**0.8159**	0.9629	0.9623

The best performance of each metric is highlighted in bold, the symbol ✗ denotes exclusion of the module, while ✓ denotes inclusion.

### 3.6 Feature visualization analysis

T-SNE technique (Van der Maaten and Hinton 2008) was further employed to visualize the low-dimensional embeddings of single-view attribute features, behavioral features, and sequence features, as well as the multi-view fused features. As shown in Fig. 5A–5C, individual feature views exhibit limited discriminative power, with unclear boundaries and significant overlap between positive and negative samples. In contrast, Fig. 5D reveals that the multi-view fused features learned by DAMFCMI form a well-separated structure in the low-dimensional space. Samples from different classes are tightly clustered with clear and distinct boundaries, indicating a strong discriminative capacity. Notably, these results highlight that DAMFCMI can effectively capture deep interaction patterns and learn the intrinsic data distribution, thereby substantially improving the separability between positive and negative samples.

**Figure 5:**
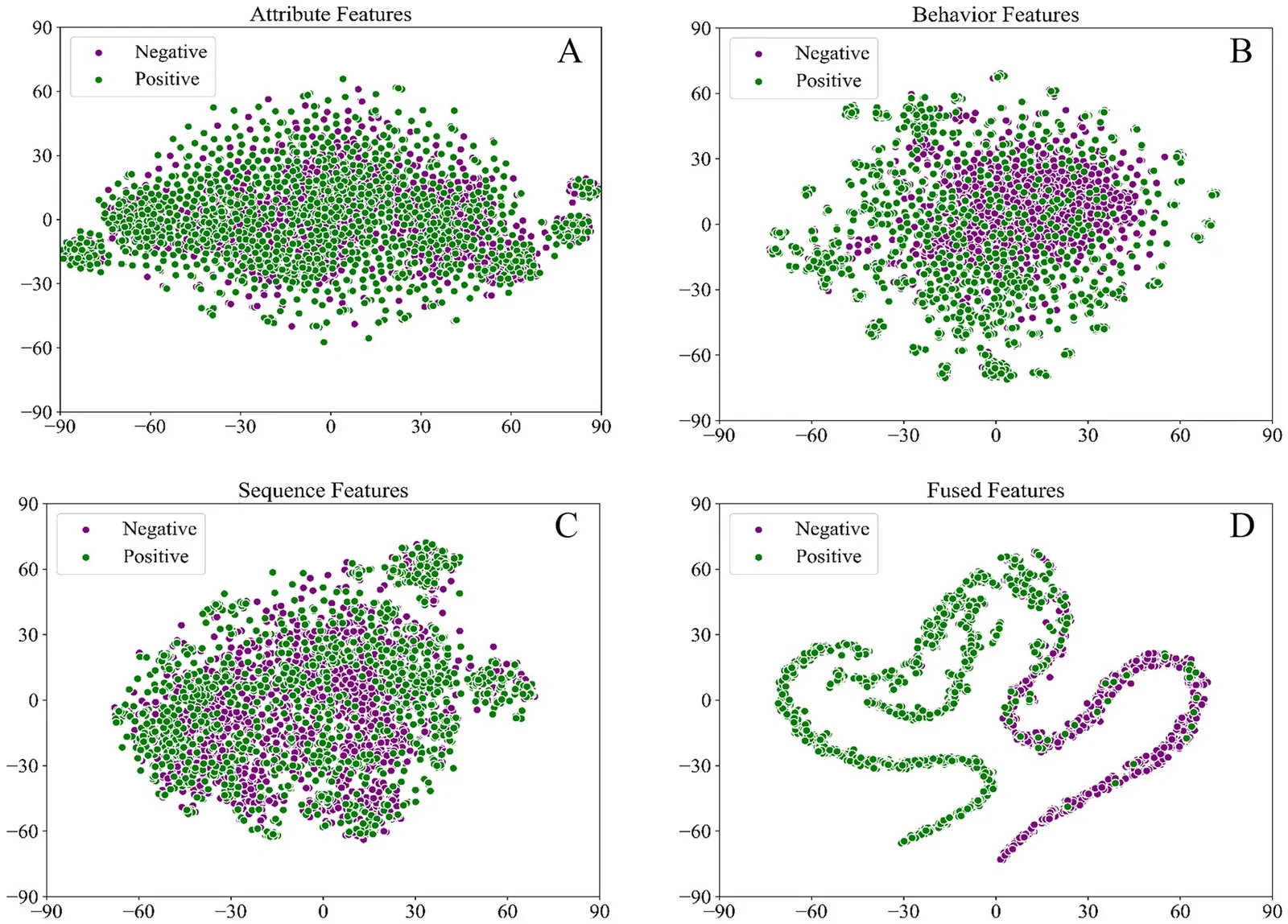
Sample distributions before and after feature fusion visualized using t-SNE.

### 3.7 Case studies

To validate the predictive capability of DAMFCMI in real-world applications, we conducted a case study focusing on circRNAs associated with cancers that have seen a significant increase in incidence in recent years, including gastric cancer, hepatocellular carcinoma, and colorectal cancer. Given the high global prevalence of these cancers and their public health impact, we randomly selected 14 circRNA entries from the CMI-9905 dataset that are known to be significantly associated with these cancers, involving 16 circRNA–miRNA interaction pairs. The results indicate that 15 out of the 16 CMI pairs were successfully predicted, with 13 of the predicted pairs corroborated by existing literature in PubMed (Table 8). For example, *hsa_circ_0092306* promotes gastric cancer development by regulating the miR-197-3p/PRKCB pathway in MKN-45 cells (Chen *et al.* 2019). hsa_circ_0103809 may facilitate the malignant development of hepatocellular carcinoma, at least in part, by regulating the miR-490-5p/SOX2 signaling pathway (Cai *et al.* 2018). hsa_circ_0079993 acts as an oncogene in colorectal cancer through the miRNA-203a-3p.1/CREB1 axis (Lu *et al.* 2019). hsa_circ_0088732 promotes glioma progression by increasing the expression of RAB3D through the miR-661 sponge mechanism (Jin *et al.* 2020). Additionally, among all the successfully predicted CMIs, only one pair of CMIs had a score lower than 0.9. These high-confidence predictions are in strong agreement with the literature, confirming that our strategy not only demonstrates robust predictive ability but also has the potential to uncover previously unknown CMI relationships of practical value. This further suggests that DAMFCMI could serve as a powerful tool for guiding relevant biological experiments.

**Table 8:** Top 15 CMIs pairs predicted by DAMFCMI of the CMI-9905 dataset.

Rank	miRNA	circRNA	PredictionScore	Evidence
1	hsa-miR-661	hsa_circ_0088732	0.99988440	PMID: 32154171
2	hsa-miR-197-3p	hsa_circ_0092306	0.99985580	PMID: 31689616
3	hsa-miR-429	hsa_circ_0084043	0.99984384	PMID: 31954160
4	hsa-miR-486-3p	hsa_circ_0092012	0.99980116	PMID: 31384171
5	hsa-miR-490-5p	hsa_circ_0103809	0.99978805	PMID: 30018710
6	hsa-miR-620	hsa_circ_0103809	0.99977744	Unconfirmed
7	hsa-miR-4302	hsa_circ_0103809	0.99976820	Unconfirmed
8	hsa-miR-335-5p	hsa_circ_0099999	0.99976170	PMID: 32154171
9	hsa-miR-331-3p	hsa_circ_0092303	0.99974895	PMID: 31182928
10	hsa-miR-338-5p	hsa_circ_0137008	0.99972620	PMID: 32158357
11	hsa-miR-490-3p	hsa_circ_0091579	0.99966950	PMID: 31841181
12	hsa-miR-1304-5p	hsa_circ_0102049	0.99963796	PMID: 31727503
13	hsa-miR-31	hsa_circ_0078710	0.99960536	PMID: 30342168
14	hsa-miR-142-5p	hsa_circ_0137439	0.97048720	PMID : 31777254
15	hsa-miR-203a-3p	hsa_circ_0079993	0.79272020	PMID: 31515467

## 4. Conclusion

In this study, we developed DAMFCMI, a hybrid-attention-guided multi-view feature fusion framework for CMI prediction. Its superior performance stems from its ability to extract complementary information from multiple feature perspectives and progressively enhance feature representations through a hierarchical fusion strategy. By jointly modeling sequence features, attribute features, and behavior features, the framework effectively mitigates the information loss often observed in single-view or shallow fusion approaches. The hybrid attention mechanism further enables deep and adaptive feature refinement. The multi-head self-attention component captures long-range dependencies within each feature view, while the cross-attention module strengthens the interactions across different feature modalities. Through this multi-level fusion strategy, DAMFCMI can identify subtle yet informative interaction signals that are frequently overlooked by traditional models. Extensive experiments and case studies show that DAMFCMI consistently outperforms existing methods in both predictive accuracy and generalization ability.

It is worth noting that although DAMFCMI was initially designed for circRNA–miRNA interaction prediction, its generalizable multi-view modeling paradigm and extensible attention-based fusion mechanism make it broadly applicable to other bioinformatics tasks. The framework can be naturally adapted to various biomolecular association prediction scenarios, such as miRNA–disease, lncRNA–protein, and drug–target interactions. Future work may incorporate heterogeneous multi-omics data—including gene expression profiles and epigenetic information—to uncover deeper layers of molecular regulation and advance our understanding of the biological mechanisms underlying complex diseases.

## Supplementary Material

vbag109_Supplementary_Data

## Data Availability

The data underlying this article are available in *GitHub* at https://github.com/yadxbiolab/DAMFCMI.
