# Supplementary material for: DAMFCMI: capturing cross-view interactions via hybrid attention for circRNA–MiRNA interaction prediction
Source: Bioinform Adv. 2026 Jul 20;6(1):vbag109. doi: 10.1093/bioadv/vbag109 (PMC13621396; doi:10.1093/bioadv/vbag109)
Supplement: Supplementary file 1 [file vbag109_supplementary_data.docx]

## Supplementary Information for

# 1. Hyperparameter Settings and Experimental Protocol

Throughout all experiments, we followed the data partitioning protocol used in existing studies (Guo, et al., 2024), where each dataset was split into training, validation, and test sets at a ratio of 8:1:1. Except for the number of heads in the multi-head self-attention module, all other training-related hyperparameters, such as the learning rate and batch size were kept at their default values. Preliminary experiments showed that changes to these parameters had little influence on model performance, so the same settings were applied consistently across all experiments. The number of attention heads was tuned on the validation set by evaluating five candidate configurations, namely 2, 4, 6, 8, and 10, and the configuration yielding the best validation performance was adopted for the final model. All performance metrics were computed and reported on the test set to ensure a strict separation between hyperparameter tuning and final evaluation, thereby guaranteeing the fairness and reproducibility of the experimental results. Detailed hyperparameter settings are provided in **Table S1**.

**Table S1.** Summary of hyperparameter settings used in the experiments.

| Category | Hyperparameter | Value/Description |
| --- | --- | --- |
| Dense Layers | Hidden units | 128 |
|  | Activation function | Relu |
|  | L2 regularization coefficient | 0.01 |
| Attention Mechanism | Number of attention heads | 8 |
|  | Key dimension | 64 |
| Feature Fusion | FFN hidden units | 128 |
|  | Fusion layer units | 64 |
| Optimizer | Optimizer | AdamW |
|  | Learning rate | 1$\times{10}^{-4}$ |
|  | Weight decay | 1$\times{10}^{-5}$ |
| Training | Batch size | 64 |
|  | Max epochs | 100 |
|  | Loss function | Binary cross-entropy |
| Callbacks | Early stopping patience | 6 |
|  | LR reduction patience | 5 |
|  | LR reduction factor | 0.2 |

# 2. Statistical stability analysis

To quantify the stability of model performance, we conducted 10 rounds of five-fold cross-validation on the CMI-20208 dataset, yielding a total of 50 independent AUC and AUPR scores. The descriptive statistics of the corresponding performance metrics are provided in Supplementary **Table S2** and **S3**.

**Table S2.** Descriptive statistics of model performance metrics on the CMI9905 dataset

| Metric | Mean | Standard Deviation | 95% Confidence Interval |
| --- | --- | --- | --- |
| AUC | 0.961661 | 0.001651 | [0.961235,0.962088] |
| AUPR | 0.961518 | 0.002529 | [0.960865,0.962171] |

**Table S3**. Student’s t-Test Results Comparing DAMFCMI with Other Models on CMI-20208

|  | P-value(two-sided) | | P-value(one-sided) | |
| --- | --- | --- | --- | --- |
| Methods | AUPR | AUC | AUPR | AUC |
| WSCD | 1.28$\times{10}^{-98}$ | 2.08$\times{10}^{-89}$ | 6.41$\times{10}^{-99}$ | 1.04$\times{10}^{-89}$ |
| KGDCMI | 1.88$\times{10}^{-97}$ | 6.06$\times{10}^{-92}$ | 9.41$\times{10}^{-98}$ | 3.03$\times{10}^{-92}$ |
| SGCCMI | 5.32$\times{10}^{-97}$ | 4.87$\times{10}^{-88}$ | 2.66$\times{10}^{-97}$ | 2.43$\times{10}^{-88}$ |
| BGF-CMAP | 2.23$\times{10}^{-91}$ | 3.56$\times{10}^{-78}$ | 1.12$\times{10}^{-91}$ | 1.78$\times{10}^{-78}$ |
| CA-CMA | 1.93$\times{10}^{-86}$ | 1.33$\times{10}^{-77}$ | 9.63$\times{10}^{-87}$ | 6.67$\times{10}^{-78}$ |
| LSAGHI | 4.95$\times{10}^{-47}$ | 1.74$\times{10}^{-45}$ | 2.48$\times{10}^{-47}$ | 8.71$\times{10}^{-46}$ |
| DGCLCMI | 8.01$\times{10}^{-40}$ | 3.93$\times{10}^{-55}$ | 4.01$\times{10}^{-40}$ | 1.96$\times{10}^{-55}$ |
| MFERL | 2.38$\times{10}^{-60}$ | 2.44$\times{10}^{-51}$ | 1.19$\times{10}^{-60}$ | 1.22$\times{10}^{-51}$ |
| MuGNetCMI | 2.44$\times{10}^{-83}$ | 1.84$\times{10}^{-74}$ | 1.22$\times{10}^{-83}$ | 9.21$\times{10}^{-75}$ |
| iHofman | 2.87$\times{10}^{-86}$ | 5.47$\times{10}^{-73}$ | 1.43$\times{10}^{-86}$ | 2.73$\times{10}^{-73}$ |

**Note**: The values shown are T-statistics and their associated p-values, computed from repeated AUC and AUPR measurements; they do not represent the AUC or AUPR performance scores themselves.

# 3. Intrinsic sequence information and physicochemical property

We extracted the intrinsic sequence information and physicochemical property features of circRNAs and miRNAs(Liu, et al., 2023), including ORF length, ORF coverage, hexamer score, Fickett score, CTD (Composition, Transition, Distribution), pI (isoelectric point), and EIIP (electron–ion interaction potential)(Klapproth, et al., 2021). ORF length refers to the nucleotide sequence between the start codon and the stop codon that can potentially be translated into a protein. It is one of the key features for evaluating the coding potential of a sequence, as sequences with strong coding potential typically possess longer ORFs(Kang, et al., 2017; Suenaga, et al., 2022). ORF coverage refers to the proportion of the longest ORF relative to the entire sequence , and is used to assess the extent to which the translatable region covers the sequence(Tong and Liu, 2019; Wang, et al., 2013). This metric helps further evaluate the coding potential of the sequence. The hexamer score is a log-likelihood ratio calculated based on the distribution of coding and non-coding RNAs in known sequences, and serves as an important feature reflecting the local coding potential of a sequence(Li and Liang, 2022; Xu, et al., 2020). The Fickett score is a classical method for evaluating the coding potential of a sequence by analyzing the nucleotide composition and their positional distribution characteristics(Fickett, 1982). CTD is a 30-dimensional feature that effectively captures the global composition, structural transition trends, and spatial distribution patterns of sequences(Govindan and Nair, 2011). It has been widely used in protein structure and function prediction due to its strong discriminative capability. The pI is one of the key indicators that can reflect differences in coding potential between circRNAs and miRNAs(Miao, et al., 2021). EIIP is also a physicochemical feature that reveals potential differences in the electronic structures of the two types of sequences(Zhou and Pang, 2018).

# 4. 5-fold CV Experiments of CMI-20208、CMI-9905, and CMI-9589

To evaluate DAMFCMI on different datasets, we performed 5-fold cross-validation on three benchmark datasets: CMI-20208, CMI-9905, and CMI-9589. For each dataset, the model was trained and tested across five independent folds.**Tables S4–S6** report the detailed results for each fold on the respective datasets. Each table includes seven evaluation metrics: ACC, Sen, Spe, Prec, MCC, AUPR, and AUC. The tables provide per-fold results as well as the average across all five folds. The corresponding heatmaps are shown in **Figure S1**, illustrating the fold-wise performance across all metrics for each dataset. As shown in the figures, the evaluation results across the five folds are relatively consistent, with only slight fluctuations in individual metrics. These visualizations provide a straightforward overview of the model's stability and effectiveness on each dataset.

**Table S4.** Experimental results on the CMI-20208 dataset.

| 5-fold | ACC | Sen | Spe | Prec | MCC | AUPR | AUC |
| --- | --- | --- | --- | --- | --- | --- | --- |
| fold1 | 0.9097 | 0.9035 | 0.9159 | 0.9148 | 0.8195 | 0.9635 | 0.9633 |
| fold2 | 0.9060 | 0.8946 | 0.9174 | 0.9154 | 0.8122 | 0.9653 | 0.9641 |
| fold3 | 0.9050 | 0.8916 | 0.9184 | 0.9161 | 0.8103 | 0.9601 | 0.9602 |
| fold4 | 0.9064 | 0.8762 | 0.9366 | 0.9326 | 0.8144 | 0.9647 | 0.9625 |
| fold5 | 0.9116 | 0.9119 | 0.9114 | 0.9114 | 0.8233 | 0.9608 | 0.9615 |
| Average | 0.9077±  0.0025 | 0.8955±0.0120 | 0.9199±0.0087 | 0.9180±0.0074 | 0.8159±0.0048 | 0.9629±0.0021 | 0.9623±0.0014 |

**Table S5.** Experimental results on the CMI-9905 dataset

| 5-fold | ACC | Sen | Spe | Prec | MCC | AUPR | AUC |
| --- | --- | --- | --- | --- | --- | --- | --- |
| fold1 | 0.9359 | 0.9505 | 0.9212 | 0.9235 | 0.8721 | 0.9734 | 0.9815 |
| fold2 | 0.9323 | 0.9566 | 0.9081 | 0.9123 | 0.8657 | 0.9777 | 0.9798 |
| fold3 | 0.9338 | 0.9242 | 0.9434 | 0.9423 | 0.8678 | 0.9722 | 0.9795 |
| fold4 | 0.9364 | 0.9444 | 0.9283 | 0.9294 | 0.8728 | 0.9716 | 0.9792 |
| fold5 | 0.9293 | 0.9444 | 0.9141 | 0.9167 | 0.8590 | 0.9780 | 0.9795 |
| Avarage | 0.9335±0.0026 | 0.9440±0.0109 | 0.9230±0.0122 | 0.9248±0.0105 | 0.8674±0.0050 | 0.9746±0.0027 | 0.9799±0.0008 |

**Figure S1**: (a) are on CMI-20208; (b) are on CMI-9589; (c) are on CMI-9905.

**Table S6.** Experimental results on the CMI-9589 dataset

| 5-fold | ACC | Sen | Spe | Prec | MCC | AUPR | AUC |
| --- | --- | --- | --- | --- | --- | --- | --- |
| fold1 | 0.9307 | 0.9239 | 0.9374 | 0.9366 | 0.8614 | 0.9763 | 0.9802 |
| fold2 | 0.9296 | 0.9239 | 0.9353 | 0.9346 | 0.8593 | 0.9798 | 0.9804 |
| fold3 | 0.9312 | 0.9447 | 0.9176 | 0.9198 | 0.8627 | 0.9770 | 0.9798 |
| fold4 | 0.9369 | 0.9124 | 0.9614 | 0.9594 | 0.8749 | 0.9747 | 0.9810 |
| fold5 | 0.9337 | 0.9447 | 0.9228 | 0.9244 | 0.8676 | 0.9786 | 0.9792 |
| Avarage | 0.9324±0.0026 | 0.9299±0.0128 | 0.9349±0.0152 | 0.9349±0.0137 | 0.8651±0.0056 | 0.9773±0.0018 | 0.9801±0.0006 |

**5. Performance under Different Head Numbers**

To investigate the effect of the number of attention heads on model performance, we conducted a parameter analysis experiment by varying the number of heads from 1 to 10. While the main paper includes representative results for even-numbered settings (2, 4, 6, 8, 10), the complete results for all head numbers are provided in the supplementary table below. Each row reports the 5-fold cross-validation average across seven evaluation metrics. The experimental data are shown in **Table S7**.

**Table S7.** Hyperparameter Analysis.

| Heads | ACC | Sen | Spe | Prec | MCC | AUPR | AUC |
| --- | --- | --- | --- | --- | --- | --- | --- |
| 10 | 0.8980 | 0.8913 | 0.9046 | 0.9038 | 0.7964 | 0.9585 | 0.9593 |
| 9 | 0.8991 | 0.8886 | 0.9095 | 0.9078 | 0.7985 | 0.9591 | 0.9586 |
| 8 | 0.9077 | 0.8955 | 0.9199 | 0.9180 | 0.8159 | 0.9629 | 0.9623 |
| 7 | 0.9033 | 0.9048 | 0.9019 | 0.9027 | 0.8072 | 0.9616 | 0.9615 |
| 6 | 0.9020 | 0.8943 | 0.9097 | 0.9084 | 0.8043 | 0.9578 | 0.9581 |
| 5 | 0.9069 | 0.9109 | 0.9028 | 0.9038 | 0.8140 | 0.9605 | 0.9621 |
| 4 | 0.9014 | 0.8953 | 0.9074 | 0.9064 | 0.8029 | 0.9595 | 0.9599 |
| 3 | 0.9029 | 0.8953 | 0.9106 | 0.9093 | 0.8062 | 0.9621 | 0.9620 |
| 2 | 0.9045 | 0.9104 | 0.8986 | 0.9002 | 0.8094 | 0.9613 | 0.9615 |
| 1 | 0.9007 | 0.8936 | 0.9079 | 0.9067 | 0.8017 | 0.9606 | 0.9599 |

**6. Cold-start Evaluation Across Multiple Datasets**

To examine whether our main conclusions are sensitive to the choice of association datasets, we conducted additional cold-start experiments on three benchmark datasets: CMI-20208, CMI-9589, and CMI-9905, to systematically evaluate the stability of the proposed method across different datasets and cold-start scenarios. For each dataset, two entity-level cold-start settings were constructed, where the data were re-split and the models were re-evaluated accordingly. In the circRNA-cold setting, circRNAs in the test set were completely unseen during training, while in the miRNA-cold setting, miRNAs in the test set were not observed in the training phase. Both settings were independently applied to all three datasets, following the same experimental protocol to ensure comparability of results across datasets. We selected BGF-CMAP, DGCLCMI, and iHofman as comparison methods; these are representative approaches for circRNA–miRNA association prediction and have publicly available implementations, ensuring fair and reproducible comparisons. The experimental results are reported in Supplementary **Tables S8** and **S9**. Across all three datasets, our method consistently outperformed the competing methods in both circRNA-cold and miRNA-cold scenarios.

**Table S8**. Performance comparison under the circRNA-cold setting on three datasets.

| Datasets | CMI-20208 | |  | CMI-9589 | |  | CMI-9905 | |
| --- | --- | --- | --- | --- | --- | --- | --- | --- |
| Methods | AUPR | AUC | Methods | AUPR | AUC | Methods | AUPR | AUC |
| BGF-CMAP | 0.8913 | 0.8895 | BGF-CMAP | 0.9077 | 0.9033 | BGF-CMAP | 0.8983 | 0.8985 |
| DGCLCMI | 0.6390 | 0.6768 | DGCLCMI | 0.6191 | 0.6530 | DGCLCMI | 0.6207 | 0.5783 |
| iHofman | 0.9139 | 0.9075 | iHofman | 0.9290 | 0.9224 | iHofman | 0.9080 | 0.9051 |
| Our | 0.9290 | 0.9292 | Our | 0.9557 | 0.9607 | Our | 0.9576 | 0.9639 |

**Table S9**. Performance comparison under the miRNA-cold setting on three datasets.

| Datasets | CMI-20208 | |  | CMI-9589 | |  | CMI-9905 | |
| --- | --- | --- | --- | --- | --- | --- | --- | --- |
| Methods | AUPR | AUC | Methods | AUPR | AUC | Methods | AUPR | AUC |
| BGF-CMAP | 0.8228 | 0.8359 | BGF-CMAP | 0.8424 | 0.8499 | BGF-CMAP | 0.8557 | 0.8639 |
| DGCLCMI | 0.3859 | 0.4684 | DGCLCMI | 0.4942 | 0.4010 | DGCLCMI | 0.5331 | 0.4757 |
| iHofman | 0.8272 | 0.8148 | iHofman | 0.8949 | 0.8969 | iHofman | 0.8322 | 0.8492 |
| Our | 0.8785 | 0.8737 | Our | 0.9340 | 0.9327 | Our | 0.9377 | 0.9320 |

**REFERENCES**

Fickett JW. Recognition of protein coding regions in DNA sequences. *Nucleic Acids Res* 1982;10(17):5303-5318.

Govindan G, Nair AS. Composition, transition and distribution (CTD)—a dynamic feature for predictions based on hierarchical structure of cellular sorting. In**:** 2011 IEEE International Conference on Bioinformatics and Biomedicine (BIBM). IEEE; 2011:1-6.

Guo LX, Wang L, You Z-H et al. Biolinguistic graph fusion model for circRNA–miRNA association prediction. *Brief Bioinform* 2024;25(2):bbae058.

Kang YJ, Yang DC, Kong L et al. CPC2: A fast and accurate coding potential calculator based on sequence intrinsic features. *Nucleic Acids Res* 2017;45(W1):W12-W16.

Klapproth C, Sen R, Stadler PF et al. Common features in lncRNA annotation and classification: A survey. *Non-Coding RNA* 2021;7(4):77.

Li M, Liang C. LNCDC: A machine learning-based tool for long non-coding RNA detection from RNA-seq data. *Sci Rep* 2022;12(1):19083.

Liu T, Zou B, He M et al. LncReader: Identification of dual functional long noncoding RNAs using a multi-head self-attention mechanism. *Brief Bioinform* 2023;24(1):bbac563.

Miao Q, Ni B, Tang J. Coding potential of circRNAs: new discoveries and challenges. *PeerJ* 2021;9:e10718.

Suenaga Y, Kato M, Nagai M et al. Open reading frame dominance indicates protein‐coding potential of RNAs. *EMBO Rep* 2022;23(6):e54321.

Tong X, Liu S. CPPred: Coding potential prediction based on the global description of RNA sequence. *Nucleic Acids* *Res* 2019;47(8):e43.

Wang L, Park HJ, Dasari S et al. CPAT: Coding-potential assessment tool using an alignment-free logistic regression model. *Nucleic Acids Res* 2013;41(6):e74.

Xu Y, Zhao X, Liu S et al. Predicting long non-coding RNAs through feature ensemble learning. *BMC Genomics* 2020;21:1-12.

Zhou HX, Pang X. Electrostatic interactions in protein structure, folding, binding, and condensation. *Chem Rev* 2018;118(4):1691-1741.
